# Thalamotemporal alteration and postoperative seizures in temporal lobe epilepsy

**DOI:** 10.1002/ana.24376

**Published:** 2015-03-13

**Authors:** Simon S. Keller, Mark P. Richardson, Jan‐Christoph Schoene‐Bake, Jonathan O'Muircheartaigh, Samia Elkommos, Barbara Kreilkamp, Yee Yen Goh, Anthony G. Marson, Christian Elger, Bernd Weber

**Affiliations:** ^1^ Department of Molecular and Clinical PharmacologyInstitute of Translational MedicineUniversity of LiverpoolLiverpoolUnited Kingdom; ^2^ Department of RadiologyWalton Centre National Health Service Foundation TrustLiverpoolUnited Kingdom; ^3^ Department of Clinical NeuroscienceInstitute of PsychiatryKing's College LondonLondonUnited Kingdom; ^4^ Department of EpileptologyUniversity of BonnBonnGermany; ^5^ Department of Neurocognition/ImagingLife and Brain Research CenterBonnGermany; ^6^ Department of NeuroimagingInstitute of Psychiatry, King's College LondonLondonUnited Kingdom

## Abstract

**Objective:**

There are competing explanations for persistent postoperative seizures after temporal lobe surgery. One is that 1 or more particular subtypes of mesial temporal lobe epilepsy (mTLE) exist that are particularly resistant to surgery. We sought to identify a common brain structural and connectivity alteration in patients with persistent postoperative seizures using preoperative quantitative magnetic resonance imaging and diffusion tensor imaging (DTI).

**Methods:**

We performed a series of studies in 87 patients with mTLE (47 subsequently rendered seizure free, 40 who continued to experience postoperative seizures) and 80 healthy controls. We investigated the relationship between imaging variables and postoperative seizure outcome. All patients had unilateral temporal lobe seizure onset, had ipsilateral hippocampal sclerosis as the only brain lesion, and underwent amygdalohippocampectomy.

**Results:**

Quantitative imaging factors found not to be significantly associated with persistent seizures were volumes of ipsilateral and contralateral mesial temporal lobe structures, generalized brain atrophy, and extent of resection. There were nonsignificant trends for larger amygdala and entorhinal resections to be associated with improved outcome. However, patients with persistent seizures had significant atrophy of bilateral dorsomedial and pulvinar thalamic regions, and significant alterations of DTI‐derived thalamotemporal probabilistic paths bilaterally relative to those patients rendered seizure free and controls, even when corrected for extent of mesial temporal lobe resection.

**Interpretation:**

Patients with bihemispheric alterations of thalamotemporal structural networks may represent a subtype of mTLE that is resistant to temporal lobe surgery. Increasingly sensitive multimodal imaging techniques should endeavor to transform these group‐based findings to individualize prediction of patient outcomes. Ann Neurol 2015;77:760–774

Pharmacoresistant epileptic seizures are strongly associated with mesial temporal lobe epilepsy (mTLE) due to hippocampal sclerosis (HS).^1^, ^2^ mTLE is the epilepsy disorder for which operations are the most frequently performed.^3^ Although surgical treatment of intractable mTLE is very effective, producing a 7‐fold increased likelihood of seizure freedom and significantly improved quality of life 1 year after surgery compared to an unoperated control group,^4^ 30 to 40% of patients will continue to experience disabling postoperative seizures 2 years after surgery.^4^, ^5^ The proportion of patients continuing to experience any seizure‐related symptom (eg, aura, nondisabling seizures) is greater, particularly after longer periods after surgery.^6^ It is currently unknown why a large subgroup of patients continues to experience seizures despite surgical intervention. Several studies have reported associations between various clinical factors and postoperative outcome in mTLE,^3^, ^6^ whereas other studies have reported no associations with the same clinical variables.^7^, ^8^ Furthermore, given that different clinical associations with seizure outcome have been reported at different postoperative time points in the same patients,^3^ and given the clinical heterogeneity of mTLE, preoperative clinical data do not allow reliable prediction of likely outcome for individual patients.

Patients with mTLE and evidence of a focal lesion, such as HS, are known to have improved postoperative outcome compared to patients with mTLE and no lesion.^7^, ^9^ However, up to 40% of patients with mTLE and HS will continue to experience persistent postoperative seizures.^9^ There has therefore been an attempt in some studies to identify brain alterations, most typically increased atrophy from T1‐weighted MRI, in patients with continued seizures relative to those rendered seizure free. There are, however, inconsistent findings. Some studies have indicated that hippocampal volume contralateral to the seizure focus may be abnormal in patients with persistent postoperative seizures,^10^, ^11^ whereas others have not.^12^, ^13^, ^14^ More morphometric MRI research on postoperative prognosis is required. Given the recent modifications in the classification of epilepsy disorders to consider the importance of brain networks involved in seizure onset, including focal epilepsies,^15^ there has been a new direction of research in mTLE to model neuroimaging data in terms of connected networks.^16^ An increasing volume of research is indicating that mTLE is a systems network disorder.^16^, ^17^, ^18^ It is conceivable that structural and functional network alterations are not uniform in mTLE, and specific subtypes of network alterations may be resistant to pharmacological treatment and surgical intervention.

Investigation of the relationship between preoperative brain connectivity and postoperative seizure outcome in patients with mTLE using diffusion tensor imaging (DTI) is lacking, and may provide important insights into the contribution of brain alterations to persistent seizures that cannot be visualized or quantified using conventional MRI data routinely acquired in the context of preoperative evaluation. In the present study, we performed a series of volumetric, morphometric, and DTI investigations assessing the relationship between preoperative quantitative imaging and postoperative seizure outcome. We additionally investigated the relationship between the extent of mesial temporal lobe resection and postoperative outcome by quantitatively analyzing postoperative MRI data.

## Subjects and Methods

### Participants

We studied 87 patients with mTLE and radiological evidence of unilateral HS (mean age = 39.4 years, standard deviation [SD] = 13.4; 55 left mTLE, 32 right mTLE) who underwent preoperative MRI scanning, amygdalohippocampectomy, and postoperative follow‐up at University Hospital Bonn, Germany. As per standardized protocol, each patient had a detailed clinical assessment to ascertain seizure semiology, interictal electroencephalogram (EEG), long‐term video EEG monitoring, if clinically necessary additional invasive electrophysiological investigations, MRI (T1 weighted, T2 weighted, and fluid‐attenuated inversion recovery [FLAIR]), and neuropsychological assessment.^19^ HS was identified by an expert neuroradiologist with considerable experience in lesion diagnosis in epilepsy, which was defined by hippocampal volume loss and internal structure disruption on T1‐weighted scans, and/or hyperintensities on T2‐weighted and FLAIR images. There was no evidence of bilateral HS in any patient, all patients had seizures of presumed unilateral temporal lobe origin, and there was no evidence of a secondary extrahippocampal lesion that may have contributed to seizures. All patients underwent standardized amygdalohippocampectomy^20^ and routine diagnostic analysis of resected hippocampal specimens by an experienced neuropathologist. Postsurgical seizure outcome was assessed using the International League Against Epilepsy (ILAE) outcome classification system.^21^ All patients had a minimum of 1 year and an average of 2 years of postoperative follow‐up. We also recruited a sample of 80 age‐ and sex‐matched neurologically and psychiatrically healthy controls (mean age = 40.0 years, SD = 13.2), who were scanned on the same MRI system using the same magnetic resonance sequences. All patients and controls provided written informed consent, and the local ethics committee approved this study.

### MRI Acquisition

All study participants underwent MRI at the Life and Brain Center in Bonn on a 3T scanner (Magnetom Trio; Siemens, Erlangen, Germany). An 8‐channel head coil was used for signal reception. We acquired T1‐weighted magnetization‐prepared rapid acquisition gradient echo (MPRAGE) images (160 slices, repetition time [TR] = 1,300 milliseconds, inversion time [TI] = 650 milliseconds, echo time [TE] = 3.97 milliseconds, resolution = 1.0 × 1.0 × 1.0mm, flip angle = 10°, acquisition time = approximately 7 minutes) for all controls and all patients prior to surgery. We also acquired postoperative T1‐weighted MPRAGE images for 52 patients. Furthermore, diffusion‐weighted data (diffusion‐weighted single shot spin‐echo echo planar imaging sequence, TR = 12 seconds, TE = 100 milliseconds, 72 axial slices, resolution = 1.726 × 1.726 × 1.7mm, no cardiac gating, GRAPPA acceleration factor = 2.0) was acquired for 46 patients (preoperatively) and 40 controls within the same scanning session. Diffusion gradients were equally distributed along 60 directions (b‐value = 1,000 s/mm^2^). Additionally, 7 data sets with no diffusion weighting (b‐value = 0 s/mm^2^) were acquired initially and interleaved after each block of 10 diffusion‐weighted images.

### Analysis: Structural MRI

The T1‐weighted data were analyzed using 3 approaches: (1) preoperative volumetric analysis of the hippocampus and entorhinal cortex; (2) preoperative voxel‐based morphometry (VBM) for analysis of whole brain atrophy; and (3) delineation of surgical lacunae, and extent of resection of mesial temporal structures, on postoperative MRIs.

We performed rigorous region‐of‐interest (ROI) manual volumetry of the left and right hippocampus and entorhinal cortex, which are brain structures considered to be of crucial importance for the generation of mesial temporal lobe seizures. We chose to use rater‐dependent manual techniques given their increased reliability over automated techniques generally, and also given the particular difficulties in reliable automated segmentation of the mesial temporal lobe into its constituent regions, particularly the entorhinal cortex. We quantified hippocampal volume using our previous described and well‐validated stereological approach.^22^ Entorhinal volume was estimated based on published anatomical boundaries^23^ using 3D Slicer software (http://www.slicer.org). All volumetric data were acquired blind to participant ID. We also determined whole (global) cortical and white matter volumes, which were automatically generated using FreeSurfer software (v5.3.0; http://surfer.nmr.mgh.harvard.edu). All volumetric data were generated in participant native space, and analyzed with other extracted ROI data (see statistical analysis below).

We performed VBM^24^ techniques in context of the VBM8 toolbox (http://dbm.neuro.uni‐jena.de/vbm/) running in SPM8 (http://www.fil.ion.ucl.ac.uk/spm/software/spm8). For ease of interpretation, the images of patients with right mTLE (37% of the patient sample) were flipped left to right so that all data could be analyzed together and treated as ipsilateral and contralateral to seizure onset/intended surgery, as undertaken previously.^10^, ^25^, ^26^ To minimize left‐to‐right bias when comparing with controls, we also side‐flipped 37% (n = 30) of the healthy control sample (which was also performed for the above volumetric approaches). Images were bias‐corrected, tissue classified, and spatially normalized using default VBM8 parameters, including the DARTEL algorithm, which increases the accuracy of the alignment between images.^27^ Each data set was modulated to correct for regional alterations in brain structure caused by the normalization process. Optimally processed gray matter images were smoothed with a 10mm full‐width at half‐maximum isotropic Gaussian kernel, and compared between healthy controls, patients surgically rendered free from all seizure‐related symptoms (ILAE I), and patients continuing to experience persistent postoperative seizures (ILAE II–VI) on a voxel‐by‐voxel basis using the general linear model. Only results surviving multiple whole brain corrections using the familywise error rate (*p* < 0.05) are reported, following previous recommendations for groupwise analyses in mTLE.^28^

To determine whether postoperative outcome was related to the total extent of temporal lobe resection, we manually delineated each surgical lacuna on postoperative T1‐weighted images using a previously described approach.^29^ We generated resection ROIs using FSL software (v5.0.4; http://fsl.fmrib.ox.ac.uk/fsl/fslwiki), from which resection volume in native space was determined. We additionally spatially registered each resection map to standard space using FSL tools described below and employed a lesion mapping technique^29^ so that resection maps could be generated and permit visualization of the differences in resection extent between patient outcome groups. To remain consistent with morphometric analyses, right‐sided lacuna labels were side‐flipped and all resections were considered together as ipsilateral for each outcome group. To determine whether the extent of resection of individual mesial temporal lobe structures was related to outcome, we coregistered postoperative MRIs and corresponding surgical lacuna masks to each patient's preoperative MRI using linear registration techniques in FSL software (FLIRT, http://fsl.fmrib.ox.ac.uk/fsl/fslwiki/FLIRT), and calculated the volume of intersection between the lacuna mask and labels of the hippocampus, amygdala, and entorhinal cortex generated from preoperative MRIs. Entorhinal labels were generated from manual volume measurements described above, where hippocampal and amygdala labels were generated in each patient's native space using FreeSurfer software, with manual edits to ensure that only hippocampal and amygdala tissue was labeled. Data were expressed as percentage of each structure resected.

### Analysis: Diffusion Tensor Imaging

The goal of DTI analyses was to investigate whether the anatomical abnormalities in patients with persistent seizures identified in VBM analyses were related to connectivity with the epileptogenic hippocampus. DTI analyses were performed in the context of Functional MRI of the Brain (FMRIB)'s Diffusion Toolbox for probabilistic tractography running in FSL. Prior to probabilistic tracking of fibers, DTI data were preprocessed by (1) correcting for the effects of eddy currents; (2) brain extracting a non–diffusion‐weighted volume using the Brain Extraction Tool; (3) reconstructing the diffusion tensors using DTIFIT; (4) determining the diffusion parameters at each voxel, using bedpostX; and (5) generating transformation matrices from nonlinearly registered DTI data to spatially normalized space (MNI152), as previously described.^17^ Following preprocessing, probtrackX was used for probabilistic tractography using waypoint masks, which necessitates the use of a seed mask and target mask. In these analyses, only streamlines that pass through both seed and target are included in the calculation of the connectivity distribution; those that pass through neither or just one mask are discarded. In the present study, we used the region of the brain found to be abnormal using VBM in patients with persistent seizures relative to those rendered seizure free as the seed region, and the ipsilateral and contralateral hippocampus (separately) as the target. The abnormal cluster of voxels obtained from VBM was exported from SPM8, transformed into MNI152 space using FMRIB's Nonlinear Image Registration Tool (FNIRT), and binarized. Hippocampal targets for each participant were obtained from the automated hippocampal labeling algorithms incorporated into FreeSurfer software, which were registered into standard space using FNIRT. For each participant, the number of connectivity streamlines that passed through both the seed and hippocampal target were thresholded at a value of 100 to minimize the effects of nonspecific connections and image noise, as previously described.^30^ Probabilistic tracts for each individual were binarized, from which mean fractional anisotropy (FA) and mean diffusivity (MD) values were derived using FSL utility tools. To determine whether any pathway differences between groups were specific to the probabilistic paths or were a reflection of global FA and MD differences, we compared mean FA and MD between outcome groups in an anatomically nonspecific ROI cube (10 mm^3^) located in the ipsilateral dorsomedial frontal region, which is a brain area not expected to be related to postoperative outcome. Mean FA and MD of this control region and probabilistic pathways in the ipsilateral and contralateral hemisphere were compared between patient outcome groups and healthy controls.

### Statistical Analysis

Analyses of clinical, volumetric, resection, and DTI data were performed using SPSS (v21.0; IBM SPSS Statistics, Armonk, NY). The relationship between outcome and categorical data (incidence of childhood febrile convulsions, secondary generalized tonic–clonic seizures [SGTCS], and meningitis, gender, side of surgery, and type of access for amygdalohippocampectomy) was investigated using chi‐square tests. Group differences in hippocampal and entorhinal volume, global cortical and white matter volume, resection volume (raw and corrected for hemispheric volume), and mean FA and MD of probabilistic tracts and of the control region in the dorsomedial frontal lobe were determined using a univariate analysis of variance (ANOVA), including a post hoc Bonferroni analysis to resolve directionality and correct for multiple comparisons. Linear regressions were used to investigate relationships between imaging data and clinical variables.

## Results

### Clinical Data

Forty‐seven (54%) patients were surgically rendered free from any seizure‐related symptom (ILAE I; Table 1). Table 2 presents the preoperative clinical data with respect to postoperative outcome for the whole group of patients. Patients with persistent postoperative seizures had a significantly higher incidence of preoperative SGTCS relative to patients rendered seizure free. There were no other significant differences between outcome groups with respect to clinical variables.

**Table 1:** Postoperative Outcome Classification

ILAE Outcome Classification	No. (%)
I	47 (54.0)
II	7 (8.0)
III	16 (18.4)
IV	14 (16.1)
V	3 (3.4)
VI	0

ILAE = International League Against Epilepsy.

**Table 2:** Clinical Variables according to Outcome

Variable	ILAE I	ILAE II–VI	χ^2^/*F*; *p*
No.	47 (54%)	40 (46%)	—
F/M	21/26	26/14	χ^2^ = 2.82; 0.10
Age at MRI, yr [SD]	39.4 [12.6]	39.5 [14.3]	*F* = 0.002; 0.97
Age at onset of mTLE, yr [SD]	16.8 [11.8]	15.3 [12.4]	*F* = 0.33; 0.57
Presurgical duration of mTLE, yr [SD]	20.5 [12.9]	26.2 [16.1]	*F* = 2.25; 0.13
Febrile convulsions, cases	14 (29.8%)	15 (37.5%)	χ^2^ = 0.28; 0.59
Meningitis, cases	6 (12.8%)	5 (12.5%)	χ^2^ = 0.001; 0.97
Seizure frequency per month [SD]	6.6 [10.7]	8.1 [14.6]	*F* = 0.31; 0.58
SGTCS, cases	12 (25.5%)	21 (52.5%)	χ^2^ = 5.58; 0.02^a^
Age at surgery, yr [SD]	39.6 [12.5]	39.9 [14.3]	*F* = 0.01; 0.91
Side of surgery, L/R	30/19	25/13	χ^2^ = 0.05; 0.83
Follow‐up, mo [SD]	22.8 [8.2]	23.7 [9.8]	*F* = 0.33; 0.72

a Statistically significant.

F = female; ILAE = International League Against Epilepsy; L = left; M = male; MRI = magnetic resonance imaging; mTLE = mesial temporal lobe epilepsy; R = right; SD = standard deviation; SGTCS = secondary generalized tonic–clonic seizures.

### Resection

**Figure 1:**
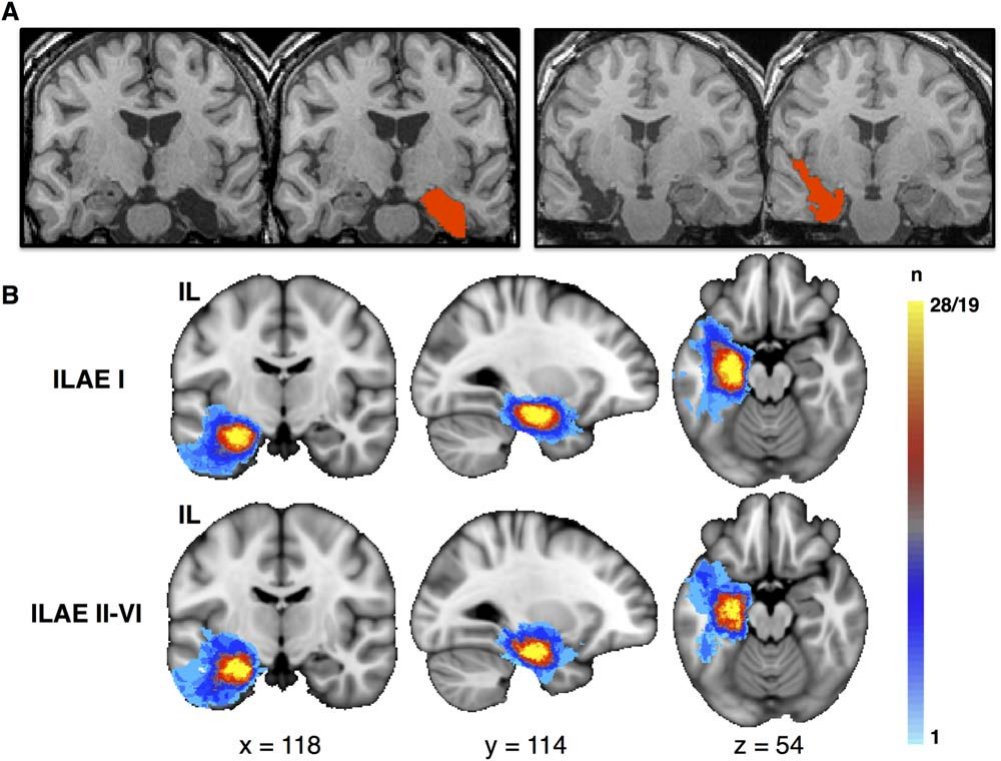
Resection and postoperative outcome. (A) Postoperative T1‐weighted magnetic resonance imaging of a left amygdalohippocampectomy via subtemporal access (left) and a right amygdalohippocampectomy via transsylvian access (right). The red area indicates the manually delineated surgical lacuna from which volumes were determined and resection mapping was performed. (B) Resection load in standard space according to postoperative outcome. Voxels are color‐coded according to the number of times a given voxel was included in the surgical lacuna (light blue, voxel was included in resection in 1 patient; yellow, voxel was included in resection in all patients within each group). There are no obvious visual differences between lesion extent and outcome, which was confirmed by statistical analysis of individual resection volumes (see main text). IL = ipsilateral; ILAE = International League Against Epilepsy; n = number of patients. [Color figure can be viewed in the online issue, which is available at www.annalsofneurology.org.]

Amygdalohippocampectomy was performed using a subtemporal (n = 41; 24 seizure free/17 postoperative seizures), transsylvian (n = 40; 26/14), or transcortical (n = 6; 5/1) approach. There was no significant categorical relationship between type of amygdalohippocampectomy access and outcome (chi‐square = 1.49, *p* = 0.48). All patients had histopathological evidence of HS on resected specimens, as determined by an experienced neuropathologist. Of the 87 patients for whom we analyzed preoperative MRI data, 52 had postoperative T1‐weighted scans for analysis, 47 of which were usable for quantitative analysis (movement artifact prohibited analysis in 5 patients). Of these patients, 28 were rendered seizure free and 19 continued to experience postoperative seizures. Resection maps are shown in Figure 1. There was no statistically significant difference between outcome groups in surgical lacuna volume or the extent of resection of individual mesial temporal structures (Table 3). There were, however, trends for an increased resection volume of the entorhinal cortex (*p* = 0.10) and amygdala (*p* = 0.07) in patients rendered seizure free relative to those with persistent seizures.

**Table 3:** Extent of Resection Data according to Group

Variable	ILAE I, n = 28, 60%	ILAE II–VI, n = 19, 40%	*F*	*p*
Surgical lacuna: raw volume, mm^3^	12,762 (5,680)	11,158 (5,192)	1.2	0.28
Surgical lacuna: normalized, mm^3^	2.69 (1.5)	2.43 (1.0)	1.1	0.32
Hippocampal resection, %	51.07 (13.51)	48.11 (12.49)	0.52	0.48
Amygdala resection, %	56.51 (22.27)	42.62 (25.81)	3.46	0.07
Entorhinal resection, %	66.18 (23.89)	54.01 (22.86)	2.72	0.11

Values are mean (standard deviation).

ILAE = International League Against Epilepsy.

### MRI: Volumetry

**Figure 2:**
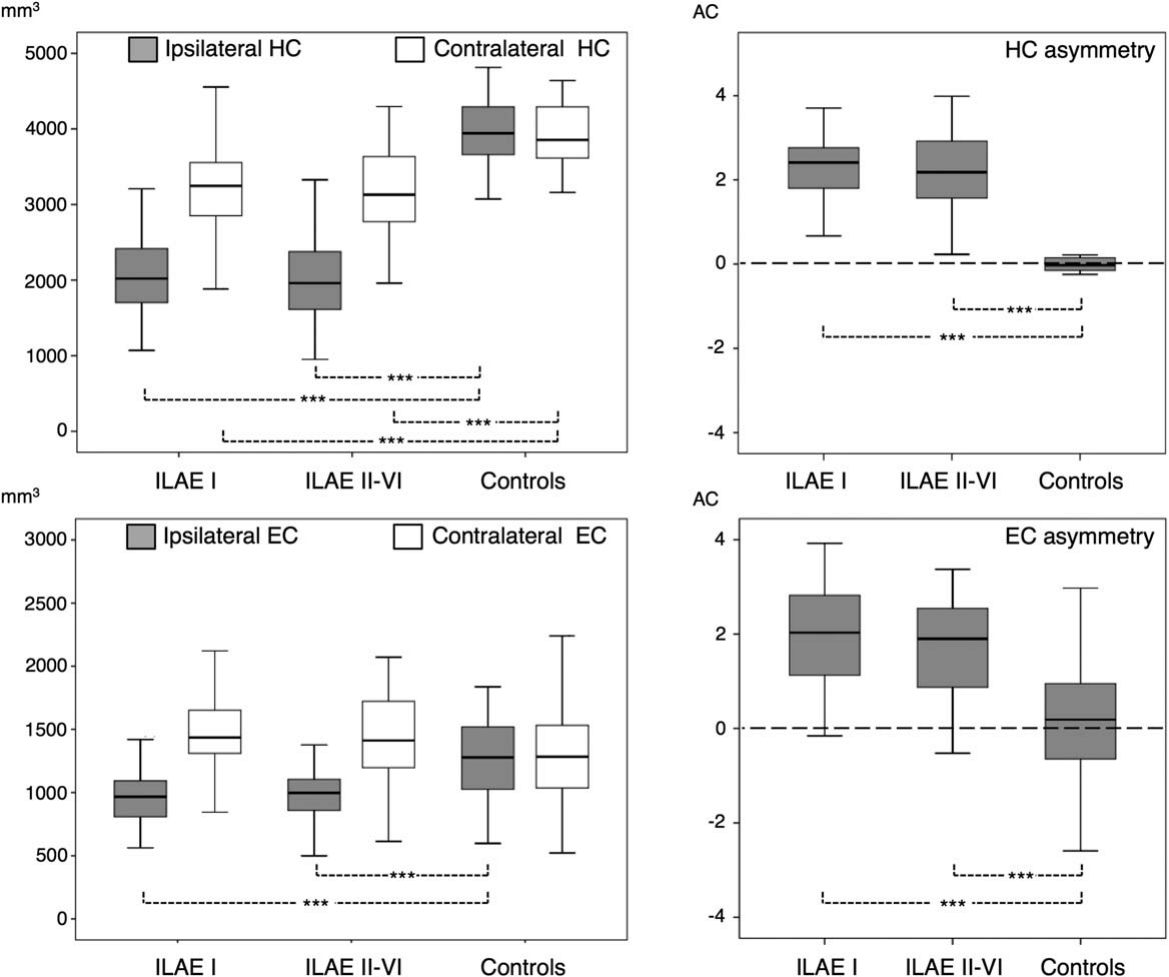
Hippocampal (HC) and entorhinal cortex (EC) volume differences between patient outcome groups and healthy controls. Significant differences are observed between patients and controls, but not between patient outcome groups. AC = asymmetry coefficient; ILAE = International League Against Epilepsy. ***p < 0.001.

There was a significant main effect of group (controls, ILAE I patients, and ILAE II–VI patients) on ipsilateral hippocampal (*F* = 143.4, *p* < 0.001), entorhinal cortex (*F* = 15.7, *p* < 0.001), and whole cortical (*F* = 15.1, *p* < 0.001) volume, contralateral hippocampal (*F* = 24.5, *p* < 0.001) and whole cortical (*F* = 8.8, *p* < 0.001) volume, and hippocampal (*F* = 82.6, *p* < 0.001) and entorhinal (*F* = 5.4, *p* = 0.005) volume asymmetry. Post hoc analysis with Bonferroni correction revealed that patients rendered seizure free and those with continuing seizures had significantly reduced ipsilateral hippocampal, entorhinal, and whole cortical volume, contralateral hippocampal and whole cortical volume, and hippocampal and entorhinal volume asymmetry relative to controls (all *p* < 0.001; Fig 2, Table 4). Bilateral volume reduction of the hippocampus in patient outcome groups was asymmetrical, with ipsilateral volume reduction being substantially greater than contralateral volume reduction. There were no significant differences in ipsilateral or contralateral hippocampal or entorhinal volume, or volume asymmetry, between outcome groups (see Fig 2). Measures of whole cortical or white matter volume also did not differ between outcome groups. There were no significant correlations between volumetric and clinical data.

**Table 4:** Mean (Standard Deviation) of Volumetric Data according to Group

Variable	Side	ILAE I, n = 47, 54%	ILAE II–VI, n = 40, 46%	Controls
Hippocampus	Ipsilateral	2,088.2 (654.6)	2,046.8 (570.8)	3,946.9 (427.1)
	Contralateral	3,191.9 (650.5)	3,152.3 (581.0)	3,943.4 (404.0)
Entorhinal	Ipsilateral	1,001.6 (272.5)	995.6 (240.0)	1,265.6 (330.3)
	Contralateral	1,484.1 (354.0)	1,425.4 (368.1)	1,320.1 (391.2)
Cortex	Ipsilateral	231,179.2 (34,379.8)	223,708.6 (34,110.8)	254,711.1 (27,208.0)
	Contralateral	235,804.6 (35,270.5)	227,466.5 (34,923.0)	252,709.0 (27,106.9)
White matter	Ipsilateral	238,488.3 (36,320.9)	234,621.6 (33,543.2)	243,514.7 (30,375.5)
	Contralateral	243,844.0 (36,622.0)	238,899.0 (33,612.0)	244,777.0 (29,794.0)

Volumes are in mm^3^.

ILAE = International League Against Epilepsy.

We additionally analyzed data separately for patients who underwent left‐ and right‐sided surgery. In patients undergoing a left‐sided resection and controls, there was a significant main effect of outcome group (ILAE I, ILAE II–VI, controls) on preoperative left hippocampal (*F* = 56.2, *p* < 0.001), left entorhinal cortex (*F* = 18.0, *p* < 0.001), and right hippocampal (*F* = 7.2, *p* = 0.001), but not right entorhinal (*F* = 2.1, *p* = 0.13) volume. Post hoc analysis with Bonferroni correction revealed that left and right hippocampal and left entorhinal cortex volume was significantly reduced in left‐sided patients rendered seizure free and left‐sided patients with continuing seizures relative to controls (all *p* < 0.001), but there was no significant difference between patient outcome groups. In patients undergoing a right‐sided resection and controls, there was a significant main effect of outcome group on preoperative right hippocampal (*F* = 35.3, *p* < 0.001), left hippocampal (*F* = 5.6, *p* = 0.02), and right entorhinal cortex (*F* = 10.3, *p* < 0.001), but not left entorhinal cortex (*F* = 1.6, *p* = 0.23) volume. Post hoc testing revealed that right‐sided patients who were rendered seizure free and those with continuing postoperative seizures had significant volume reduction of the right hippocampus (both *p* < 0.001), left hippocampus (ILAE I, *p* = 0.05; ILAE II–VI, *p* = 0.03), and right entorhinal cortex (both *p* < 0.001). There was therefore no difference between left‐ and right‐sided patients in the distribution of hippocampal and entorhinal cortex volumes in relation to postoperative outcome, with both patient outcome groups exhibiting volume loss of the ipsilateral entorhinal cortex and hippocampus bilaterally relative to controls.

### MRI: Voxel‐Based Morphometry

**Figure 3:**
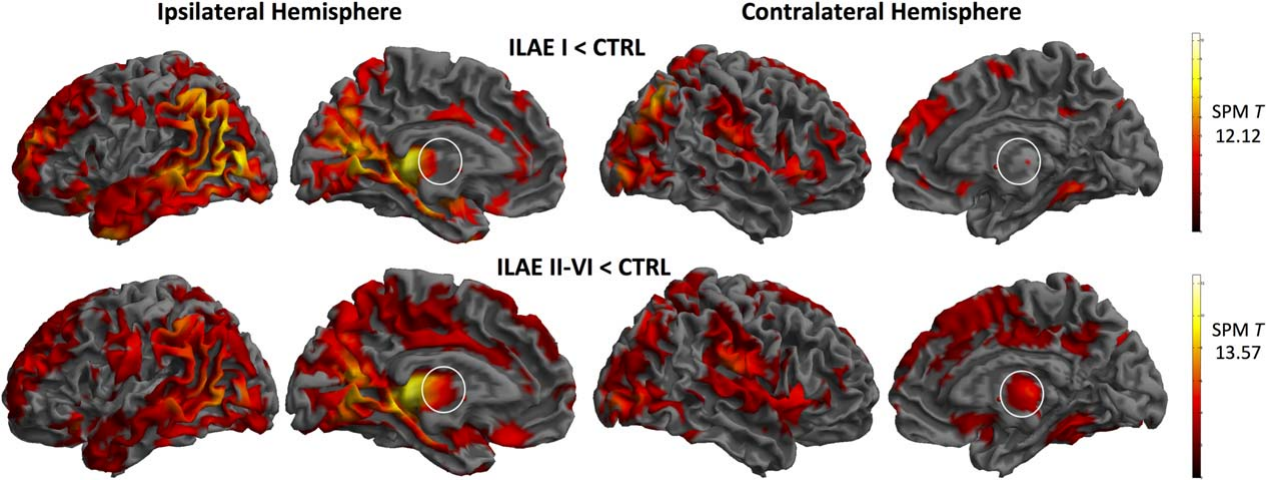
Voxel‐based morphometry results: whole brain gray matter atrophy in patient groups relative to controls. Results are difference maps (T) projected onto a standardized brain surface rendering. White circles illustrate greater general ipsilateral and contralateral thalamic atrophy in patients with persistent postoperative seizures. ILAE = International League Against Epilepsy; SPM = Statistical Parametric Mapping. [Color figure can be viewed in the online issue, which is available at www.annalsofneurology.org.]

**Figure 4:**
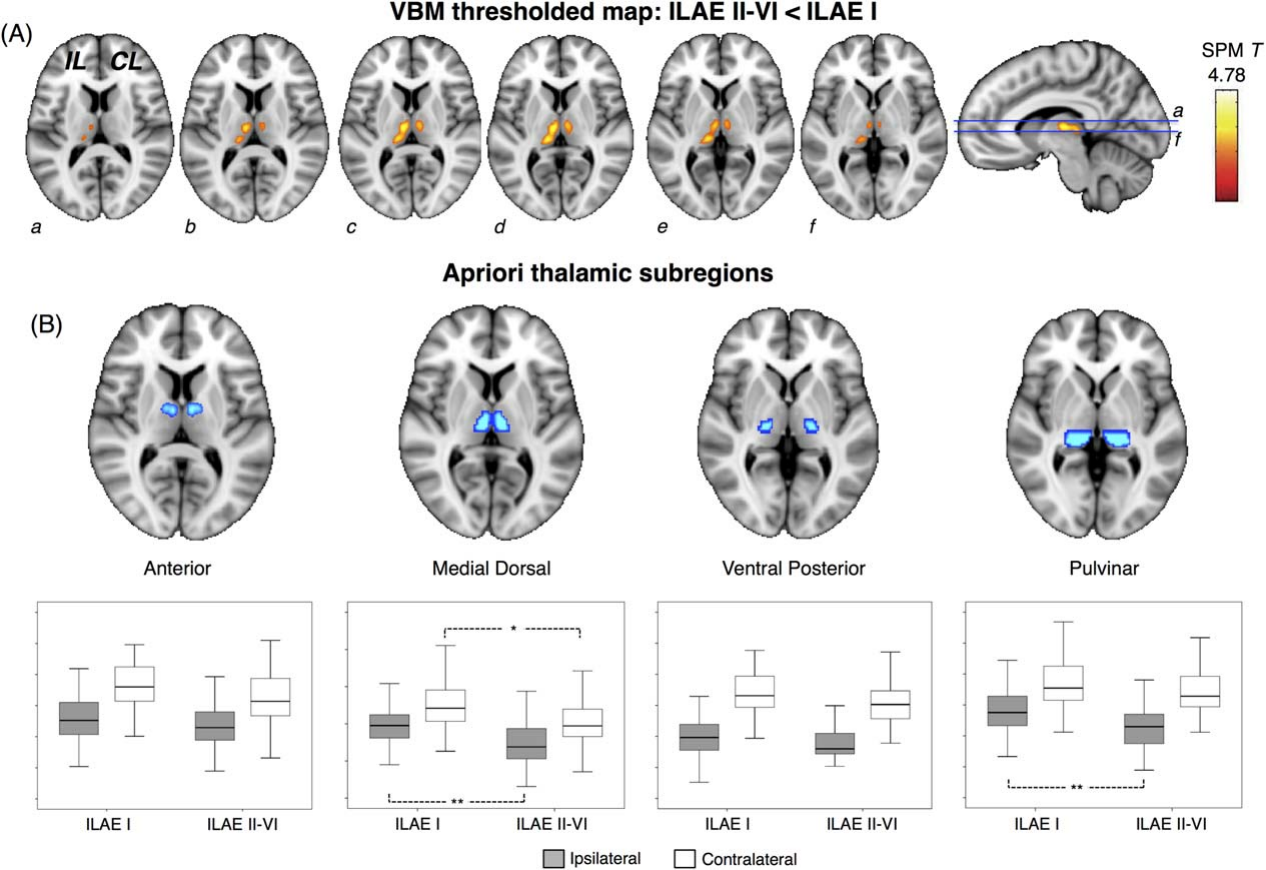
Voxel‐based morphometry (VBM) results: differences between patient outcome groups. (A) The single cluster of voxels (yellow–orange) significantly different when patient outcome groups were directly compared, which corresponded to regional thalamic atrophy in patients with persistent postoperative seizures. (B) Quantification of mean gray matter volume within 4 thalamic a priori regions of interest revealed that ipsilateral medial dorsal and pulvinar, and contralateral medial dorsal regions were significantly atrophic in patients with persistent postoperative seizures relative to those rendered seizure free. CL, contralateral; IL, ipsilateral; ILAE = International League Against Epilepsy; SPM = Statistical Parametric Mapping. *p < 0.05, **p < 0.01. [Color figure can be viewed in the online issue, which is available at www.annalsofneurology.org.]

Relative to controls, both patient outcome groups showed similar patterns of whole brain gray matter atrophy, which were most prominent in the ipsilateral hemisphere, and encompassed the mesial and lateral temporal, parietal, occipital, and frontal lobes, thalamus, and striatum (Fig 3; ILAE peak voxel, SPM T = 12.12; ILAE II–VI peak voxel, SPM T = 13.57). It was evident from these comparisons that patients with persistent postoperative seizures had more widespread and intense atrophy of the thalamus in both hemispheres (see Fig 3, circled regions). In direct comparisons between patient outcome groups, only 1 cluster of voxels was significantly different; patients with persistent seizures had significantly reduced gray matter in medial–dorsal and posterior thalamic regions relative to patients rendered seizure free. This effect was bilateral, but predominantly observed in the ipsilateral thalamus (Fig 4A; peak voxel, SPM T = 4.78; peak coordinate = −12, −15, 7).

To determine the anatomical specificity of thalamic differences between patient outcome groups, we quantified the mean voxelwise volume of the spatially normalized, segmented, and smoothed gray matter maps within 4 primary thalamic ROIs according to atlas labels^31^ constructed from the Talairach coordinate system.^32^ We chose to analyze the 4 largest subregions of the thalamus: the anterior, medial dorsal, ventral posterior, and pulvinar regions (see Fig 4B). Results were consistent with the spatial topology of thalamic differences based on whole brain VBM threshold maps, indicating that mean gray matter volume in the ipsilateral medial dorsal (ILAE I mean [SD] = 0.59 [0.10], ILAE II–VI = 0.51 [0.12]; *F* = 9.9, *p* = 0.002) and pulvinar regions (ILAE I = 0.38 [0.07], ILAE II–VI = 0.33 [0.08]; *F* = 8.4, *p* = 0.005) and the contralateral medial dorsal region (ILAE I = 0.69 [0.12], ILAE II–VI = 0.62 [0.14]; *F* = 5.6, *p* = 0.02) was significantly reduced in patients with postoperative seizures relative to those rendered seizure free (see Fig 4B). There was no significant difference between outcome groups in mean gray matter volume within ipsilateral anterior (ILAE I = 0.28 [0.04], ILAE II–VI = 0.27 [0.04]; *F* = 2.6, *p* = 0.11) and ventral posterior (ILAE I = 0.15 [0.03], ILAE II–VI = 0.14 [0.03]; *F* = 2.1, *p* = 0.15) regions or contralateral pulvinar (ILAE I = 0.46 [0.08], ILAE II–VI = 0.45 [0.08]; *F* = 1.8, *p* = 0.20), anterior (ILAE I = 0.32 [0.04], ILAE II–VI = 0.31 [0.04]; *F* = 2.1, *p* = 0.15), and ventral posterior (ILAE I = 0.22 [0.04], ILAE II–VI = 0.21 [0.04]; *F* = 1.67, *p* = 0.24) regions. Preoperative duration of mTLE was significantly correlated with mean gray matter volume in each thalamic subregion, as was chronological age in patients and healthy controls (Table 5). Thalamic changes were not significantly related to age of onset of mTLE, seizure frequency, or preoperative prevalence of secondary generalized tonic–clonic seizures.

**Table 5:** Correlations between Mean Volume of Gray Matter within the Thalamic Subregions and Duration of mTLE in Patients, and Chronological Age in Patients and Controls

Factor	Thalamic Region	*r*	*p*
Duration of mTLE	Pulvinar (I)	−0.33	0.003
	MD (I)	−0.41	>0.001
	Anterior (I)	−0.41	>0.001
	Posterior (I)	−0.24	0.03
	Pulvinar (C)	−0.23	0.04
	MD (C)	−0.40	>0.001
	Anterior (C)	−0.45	>0.001
	Posterior (C)	−0.19	0.09
Age, patient	Pulvinar (I)	−0.37	0.001
	MD (I)	−0.41	>0.001
	Anterior (I)	−0.37	0.001
	Posterior (I)	−0.20	0.08
	Pulvinar (C)	−0.33	0.002
	MD (C)	−0.40	>0.001
	Anterior (C)	−0.39	>0.001
	Posterior (C)	−0.21	0.06
Age, control	Pulvinar (L)	−0.37	0.002
	MD (L)	−0.29	0.01
	Anterior (L)	−0.29	0.01
	Posterior (L)	−0.23	0.05
	Pulvinar (R)	−0.40	>0.001
	MD (R)	−0.33	0.005
	Anterior (R)	−0.23	0.05
	Posterior (R)	−0.21	0.10

C = contralateral; I = ipsilateral; L = left; MD = medial dorsal; mTLE = mesial temporal lobe epilepsy; R = right.

### Diffusion Tensor Imaging

**Figure 5:**
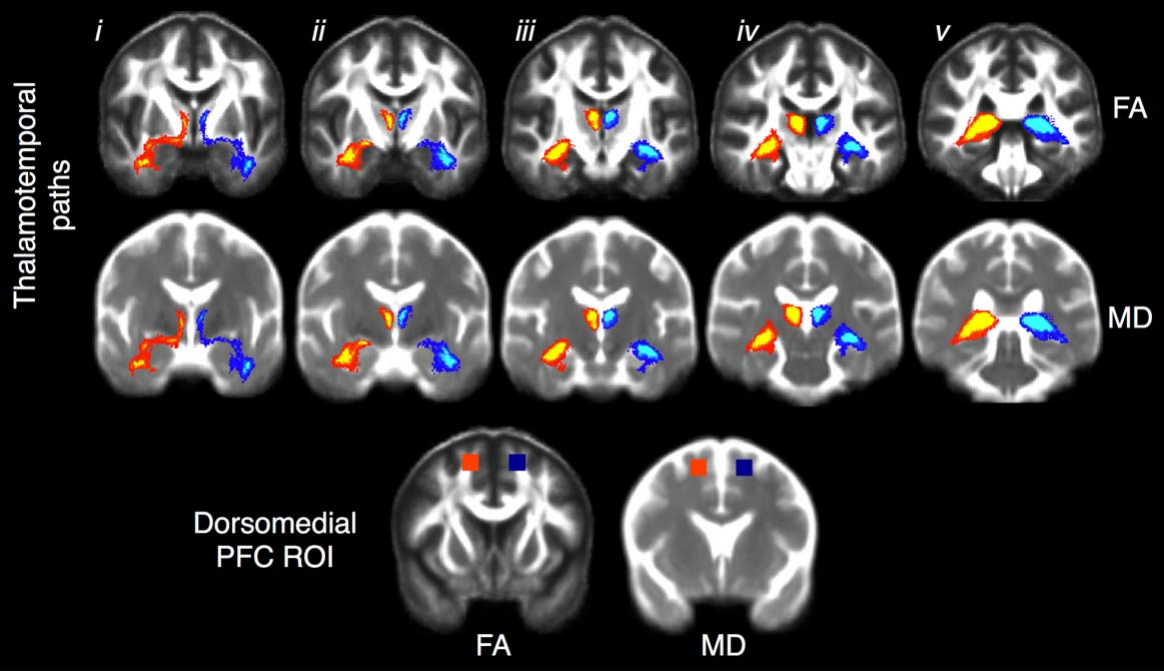
Diffusion tensor imaging probabilistic thalamotemporal paths projected onto mean fractional anisotropy (FA; top) and mean diffusivity (MD; middle) templates constructed from all patients and controls. These common paths were generated for visual purposes by averaging and thresholding (75%) the individual paths obtained from each participant in standard space. i–v indicate anterior to posterior coronal sections showing the ipsilateral (red–yellow) and contralateral (dark blue–light blue) thalamotemporal paths. The anterior sublenticular pathway can be seen in i, and the posterior fornical pathway can be seen in v. The bottom coronal sections show the control region in the dorsomedial prefrontal cortex (PFC) projected onto the same FA (left) and MD (right) template. ROI = region of interest. [Color figure can be viewed in the online issue, which is available at www.annalsofneurology.org.]

Of the 46 patients who received DTI, 21 (46%) continued to experience postoperative seizures and 25 (54%) were completely seizure free, and were therefore representative of the larger sample. Probabilistic tractography streamlines seeded from the thalamic structural abnormality and targeting the ipsilateral hippocampus revealed anterior and posterior paths between structures in all patients. These probabilistic thalamotemporal paths are shown in conjunction with the control dorsomedial prefrontal ROI in Figure 5. In particular, anterior probabilistic connections included streamlines passing from the thalamic seed, rostrally through the anterior–medial thalamus connecting with anterior regions of the mesial temporal lobe, passing underneath the lenticular nuclei, and through the temporal stem. The posterior pathway included streamlines passing from the thalamic seed, caudally through dorsomedial and pulvinar thalamic regions, and to the lateral edge of the mesial temporal lobe via the fornix and fimbria.

**Figure 6:**
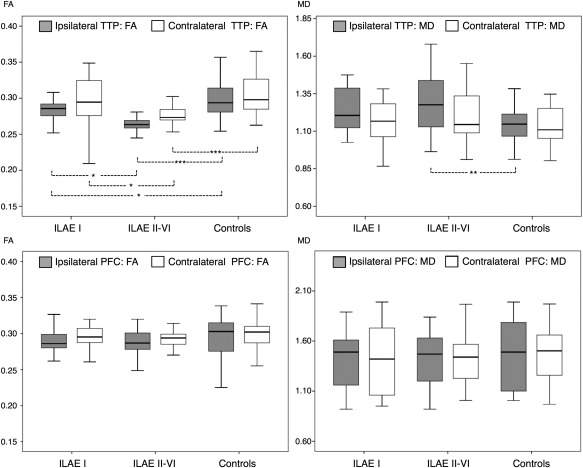
Diffusion tensor imaging results: mean fractional anisotropy (FA; left) and mean diffusivity (MD; right) of the thalamotemporal paths (TTP; top) and control region in the dorsomedial prefrontal cortex (PFC; bottom). Patients with continuing postoperative seizures show evidence of significant mean FA reduction in the ipsilateral and contralateral TTP relative to patients rendered seizure free and healthy controls (top left). Patients with a suboptimal outcome also show a significant mean MD increase in the ipsilateral TTP relative to controls (top right). No differences are observed in the control region. ILAE = International League Against Epilepsy. *p < 0.05, **p < 0.01, ***p < 0.001.

Results from comparisons of DTI data are presented in Figure 6 and Table 6. There was a significant effect of group on mean FA of the ipsilateral (*F* = 15.0, *p* < 0.001) and contralateral (*F* = 8.1, *p* = 0.001) thalamotemporal probabilistic paths, and on mean MD of the ipsilateral thalamotemporal paths (*F* = 6.7, *p* = 0.002). There was no effect of group on mean MD of the contralateral thalamotemporal paths (*F* = 2.1, *p* = 0.14), or on any DTI scalar value of the control dorsomedial region (ipsilateral FA, *F* = 0.4, *p* = 0.64; contralateral FA, *F* = 2.2, *p* = 0.12; ipsilateral MD, *F* = 0.3, *p* = 0.74; contralateral MD, *F* = 1.9, *p* = 0.15). Post hoc testing with Bonferroni correction indicated that mean FA of ipsilateral thalamotemporal paths was significantly reduced in patients who were seizure free (*p* = 0.03) and patients with persistent postoperative seizures (*p* < 0.001) relative to healthy controls, and significantly reduced in patients with persistent seizures relative to those rendered seizure free (*p* = 0.02). Furthermore, patients with persistent postoperative seizures had significant reduction of mean FA of contralateral thalamotemporal paths relative to controls (*p* < 0.001) and seizure‐free patients (*p* = 0.02), whereas there were no significant differences between the latter 2 groups. Patients with persistent seizures also had a significant increase in mean MD of the ipsilateral thalamotemporal paths relative to controls (*p* = 0.003), but not relative to patients experiencing no postoperative seizures (*p* = 0.81).

**Table 6:** Diffusion Tensor Imaging Results

Variable	Side	ILAE I, n = 25, 54%	ILAE II–VI, n = 21, 46%	Controls
Thalamotemporal paths FA	Ipsilateral	0.284 (0.024)	0.264 (0.012)	0.301 (0.030)
	Contralateral	0.300 (0.034)	0.276 (0.018)	0.306 (0.028)
Thalamotemporal paths MD	Ipsilateral	1.23 (0.14)	1.28 (0.20)	1.14 (0.13)
	Contralateral	1.16 (0.15)	1.21 (0.18)	1.13 (0.12)
PFC FA	Ipsilateral	0.287 (0.022)	0.291 (0.025)	0.293 (0.029)
	Contralateral	0.296 (0.018)	0.293 (0.020)	0.296 (0.043)
PFC MD	Ipsilateral	1.41 (0.32)	1.43 (0.26)	1.47 (0.34)
	Contralateral	1.40 (0.34)	1.40 (0.23)	1.47 (0.27)

Variables are mean FA and MD of probabilistic thalamotemporal pathways and of a nonspecific region in the dorsomedial PFC. Values are mean (standard deviation). MD values are ×1,000.

FA = fractional anisotropy; ILAE = International League Against Epilepsy; MD = mean diffusivity; PFC = prefrontal cortex.

Given that there was a trend for larger amygdala and entorhinal resections to be associated with improved outcome, we compared tractography data between patient outcome groups corrected for the extent of resection (multivariate ANOVA, resection extent treated as nuisance variable). Results indicated that patients with persistent postoperative seizures had significantly reduced FA of the ipsilateral and contralateral thalamotemporal paths when corrected for extent of resection (Table 7).

**Table 7:** Thalamotemporal Path Differences between Patient Outcome Groups Corrected for Extent of Resection

Variable	Side	Uncorrected^a^ *F* (*p*)	Lacuna *F* (*p*)	Hippocampal *F* (*p*)	Amygdala *F* (*p*)	Entorhinal *F* (*p*)
Thalamotemporal paths FA	Ipsilateral	11.72 (0.001)	5.78 (0.03)	6.27 (0.01)	4.62 (0.04)	4.47 (0.04)
	Contralateral	12.38 (0.001)	8.386 (0.003)	8.58 (0.002)	7.39 (0.009)	9.42 (0.009)
Thalamotemporal paths MD	Ipsilateral	0.97 (0.33)	0.74 (0.39)	0.90 (0.35)	0.36 (0.55)	0.41 (0.52)
	Contralateral	1.48 (0.24)	1.01 (0.20)	1.18 (0.19)	0.93 (0.34)	0.93 (0.34)

Surgical lacuna correction is volume, and hippocampal, amygdala, and entorhinal is percentage of resection.

a Discrepancies between statistical values here and in the main analysis are due to a different statistical model used; a direct comparison between patient outcome groups is used here, whereas the main analysis included controls with Bonferroni correction.

FA = fractional anisotropy; MD = mean diffusivity.

Patients with a preoperative history of SGTCS had significantly lower mean FA values of ipsilateral thalamotemporal paths relative to patients with no SGTCS (mean with SGTCS = 0.271, SD = 0.03; mean without SGTCS = 0.282, SD = 0.03; *F* = 3.67, *p* = 0.04). Mean FA of ipsilateral thalamotemporal paths was also significantly correlated with preoperative duration of mTLE (r = −0.41, *p* = 0.01). There were no other significant associations between DTI data and patient clinical information.

## Discussion

We have performed a detailed pre‐ and postoperative quantitative imaging study of postoperative seizure outcome in patients with mTLE and HS. We have observed that patients who are rendered seizure free and patients who continue to experience persistent postoperative seizures cannot be differentiated based on (1) preoperative volume of the hippocampus or entorhinal cortex, (2) global rates of preoperative brain atrophy, or (3) the extent of resection of mesial temporal lobe tissue. However, as a group, patients with persistent seizures show evidence of (1) significant atrophy of the dorsomedial and pulvinar areas of the thalamus and (2) probabilistic connection alterations between thalamus and the mesial temporal lobe, relative to both healthy controls and patients who are rendered seizure free. We discuss these findings with respect to previous studies before highlighting pertinent methodological issues.

### Quantitative Preoperative Imaging

Medial and pulvinar thalamic regions are known to be susceptible to significant neuronal loss^33^ and are important for seizure modulation and spread^34^ in animal limbic epilepsy, show ictal changes in some patients with mesial temporal seizure onset,^35^, ^36^ and have been reported to be abnormal in human structural imaging studies of mTLE.^17^, ^37^ The dorsomedial and pulvinar abnormality is part of a structural network abnormality that involves anatomical connections with the mesial temporal lobe in patients with mTLE,^17^ and we report for the first time that this abnormality is particularly prevalent in patients who continue to experience postoperative seizures. There are known anatomical pathways between the mesial temporal lobe and thalamus via the fornix (posteriorly) and the sublenticular limb of the internal capsule (anteriorly),^38^ which correspond well with the probabilistic pathways we have obtained from our DTI data (see Fig 5). It was interesting that we observed FA alterations in thalamotemporal paths ipsilateral and contralateral to the side of intended surgery in patients who went on to experience persistent postoperative seizures relative to those rendered seizure free and healthy controls, which suggests a bihemispheric network problem. Therefore, there may be a greater chance for bilateral seizure onset zones in patients with bilateral thalamic alterations who continue to experience postoperative seizures. Although we do not necessarily suggest that structural networks are epileptogenic in nature, the impairments in the thalamus and thalamic connections may contribute to a less stable functional network, which may be more amenable to ictal dynamics.^16^ It has also been reported that the extent of preoperative electrophysiological coupling between the mesial temporal lobe and thalamus is related to postoperative seizure outcome.^36^ It will be interesting to investigate how structural, functional, and metabolic alterations of thalamotemporal networks relate to alterations seen in patients with temporal plus epilepsy, which may semiologically imitate mTLE, may have significant insular involvement, and is associated with relatively poor postoperative outcomes.^39^, ^40^

Our results may provide some insight into the underlying biological mechanisms of seizure severity in mTLE. Consistent with other work,^3^, ^7^ we report that patients with continuing postoperative seizures have a greater preoperative prevalence of SGTCS relative to patients rendered seizure free. Given the importance of the thalamus in primary generalized epilepsies, it is conceivable that thalamic mechanisms play a role in secondary generalization in mTLE. However, previous work has failed to find such a relationship based on conventional volumetry of T1‐weighted MRI.^41^ We suggest this may be because of the inherent limitations of this type of imaging in identifying alterations in structural networks, given that we report an association between the preoperative experience of SGTCS and alterations of DTI probabilistic thalamotemporal paths.

The hippocampus and entorhinal cortex are generally considered to be the primary seizure generators—or primary nodes in an epileptiform network—in mTLE. However, we found no evidence for a relationship between preoperative hippocampal or entorhinal volume and postoperative outcome given that both patient groups had comparable atrophy of these structures relative to controls. Similar to our results, global hippocampal volume has been shown to be statistically unrelated to postoperative outcome in other studies.^12^, ^13^, ^14^ There have been few attempts to assess the relationship between entorhinal morphology and outcome. One study reported no association,^42^ whereas another study reported that high rates of contralateral entorhinal atrophy are related to postoperative seizure relapse.^43^ A previous study suggested that mTLE patients with persistent postoperative seizures have a more generalized distribution of abnormalities across the whole brain relative to patients who respond optimally to surgery.^25^ However, we report that global gray matter and white matter volumes were not different, and that VBM‐resolved patterns of extrathalamic gray matter atrophy were also comparable between outcome groups.

### Quantitative Postoperative Imaging

We found that there was no statistically significant relationship between the extent of mesial temporal lobe resection and postoperative seizure outcome. Although there are some articles that report such a relationship,^39^, ^44^ there are others that do not.^45^, ^46^ A review paper on temporal lobe resection and outcome reported that the extent of resection does not necessarily lead to improved postoperative seizure outcome, that patients with significant hippocampal and amygdaloidal remnants may experience excellent postoperative seizure outcomes, and that amygdalohippocampectomy and anterior temporal lobectomy do not differ in rates of seizure freedom.^47^ On the contrary, a meta‐analysis revealed that anterior temporal lobectomy was significantly more likely to result in seizure freedom relative to amygdalohippocampectomy.^48^ Another review article indicated that a second resection of mesial temporal lobe remnant tissue in patients with persistent seizures can improve outcome,^39^ which suggests that incomplete resection of mesial temporal structures may be a cause of persistent postoperative seizures. We report that there is a trend for increased resection of extrahippocampal mesial temporal structures to be related to improved outcome, but this finding does not reach statistical significance. Two previous studies reported that the extent of entorhinal and parahippocampal resection is significantly related to outcome.^49^, ^50^ The differences in findings may be due to the different approaches used to quantify resection extent. Unlike the previous studies, we delineated the boundaries of mesial temporal lobe regions on each patient's preoperative MRI and determined the intersection between these regions and the corresponding manually delineated surgical lacuna. There is intuitively more chance of resecting epileptogenic tissue if the resection is larger. However, some patients who have complete resection of mesial temporal structures continue to experience postoperative seizures,^51^ which indicates that there may be a subtype of mTLE resistant to temporal lobe resection. Patients with bihemispheric thalamotemporal connectivity alterations may represent such a subtype.

### Methodological Issues

Groupwise analyses are sensitive to common differences between groups but assume within‐group homogeneity. mTLE is a systems disorder with heterogeneous characteristics, and patients continuing to experience postoperative seizures will have heterogeneous clinical and electrophysiological contributions to a suboptimal outcome. Thalamic and thalamotemporal alterations must therefore be considered alongside other clinical and electrophysiological information. Moreover, alterations in thalamotemporal structure and connectivity as presented in this study cannot prospectively unequivocally differentiate between a patient who will develop postoperative seizures and a patient who will not, given natural neuroanatomical variability and relatively low resolution of clinically applicable MRI. Although the results of this study identify another predictive association of outcome, the overlap between seizure‐free and postoperative seizure groups is such that the findings of this study cannot be used, on their own, to predict seizure outcome after temporal lobe surgery on an individual basis. The results do, however, provide additional insight into the variability of the pathophysiology of mTLE, but it may not, for each individual, be possible to use the findings to guide surgical planning.

There are important factors not considered in this study that may hold prognostic value for outcome. The resection extent of fluorodeoxyglucose positron emission tomography (FDG‐PET) hypometabolism has been shown to be related to postoperative seizure outcome,^52^ as have atypical patterns of HS.^53^, ^54^ Our patients did not receive FDG‐PET in the context of preoperative evaluation, given that all individuals were already excellent candidates for temporal lobe surgery on the basis of semiological, electrophysiological, neuropsychological, and MRI evaluation. FDG‐PET may be used for difficult surgical cases, including those with no observable brain lesion, multiple lesions, or inconsistent epileptiform patterns on EEG. We did not perform quantitative histopathological analysis of resected hippocampal specimens to the extent of previous studies.^53^, ^54^ Quantitative analysis of hippocampal subfields using imaging to identify atypical patterns of HS represents an interesting research endeavor.^55^ However, the proportion of patients continuing to experience postoperative seizures far outweighs the proportion of patients with atypical patterns of HS, and therefore there are likely to be other significant factors that contribute to suboptimal outcome.

Finally, it is important to consider that not every patient in our series received preoperative DTI and postoperative MRI. This is because 60‐direction DTI and postoperative T1‐weighted isotropic MPRAGE acquisitions are not routinely used in clinical evaluation at our center. However, DTI and postoperative MRI data have been analyzed from a proportion of the main cohort in this study, and we do not envisage any selection bias. The outcome proportions of the subgroup of patients who received DTI are identical to the larger cohort for whom outcome data and preoperative MPRAGE data were available for analysis (ILAE I = 54%, ILAE II–VI = 46%). Given the potential significance of tractography for postoperative outcome, we now endeavor to acquire high‐quality DTI data routinely for patients who are being considered for resective surgery.

### Conclusions

It is widely considered that postoperative seizures after temporal lobe resection may be due to undetected dual pathology, contralateral relapse, extratemporal and temporal plus seizures, neocortical foci, or insufficient resection of epileptogenic tissue. However, these factors are unlikely to explain all surgical failures, and a subtype of mTLE resistant to resective surgery may exist. It is known that mTLE is a systems disorder, characterized by networked brain alterations, which may frequently involve the thalamus. These alterations may not be uniform, and differences in network alterations may explain why some patients are pharmacologically intractable and some are surgically resistant. Patients with mTLE and significant thalamotemporal network alterations may represent a subtype of mTLE particularly resistant to conventional temporal lobe surgery. Translating these groupwise analyses to inform individual patient prognosis may be an important future endeavor.

## Authorship

S.S.K., C.E., and B.W. were involved in the conception of the project. S.S.K., M.P.R., J.‐C.S.‐B., A.M., C.E., and B.W. were involved in the design of the study. J.‐C.S.‐B., C.E., and B.W. acquired the data. S.S.K., J.O., S.E., B.K., and Y.Y.G. analyzed the data. S.S.K., M.P.R., J.‐C.S.‐B., J.O., A.M., and B.W. interpreted the data. S.S.K. prepared the manuscript. All authors reviewed and edited the manuscript. S.S.K. and M.P.R. contributed equally to this article.

## Potential Conflicts of Interest

J.‐C.S.‐B.: travel expenses, Desitin, AG Praechirurgie.
